# Confirming the Diversity of the Brain after Normalization: An Approach Based on Identity Authentication

**DOI:** 10.1371/journal.pone.0054328

**Published:** 2013-01-30

**Authors:** Fanglin Chen, Longfei Su, Yadong Liu, Dewen Hu

**Affiliations:** Department of Automatic Control, College of Mechatronics and Automation, National University of Defense Technology, Changsha, Hunan, People’s Republic of China; Institution of Automation, CAS, China

## Abstract

During the development of neuroimaging, numerous analyses were performed to identify population differences, such as studies on age, gender, and diseases. Researchers first normalized the brain image and then identified features that represent key differences between groups. In these studies, the question of whether normalization (a pre-processing step widely used in neuroimaging studies) reduces the diversity of brains was largely ignored. There are a few studies that identify the differences between individuals after normalization. In the current study, we analyzed brain diversity on an individual level, both qualitatively and quantitatively. The main idea was to utilize brain images for identity authentication. First, the brain images were normalized and registered. Then, a pixel-level matching method was developed to compute the identity difference between different images for matching. Finally, by analyzing the performance of the proposed brain recognition strategy, the individual differences in brain images were evaluated. Experimental results on a 150-subject database showed that the proposed approach could achieve a 100% *identification ratio*, which indicated distinct differences between individuals after normalization. Thus, the results proved that after the normalization stage, brain images retain their main distinguishing information and features. Based on this result, we suggest that diversity (individual differences) should be considered when conducting group analysis, and that this approach may facilitate group pattern classification.

## Introduction

The brain is the center of the nervous system and the most important and complex organ in the human body. In neuroscience, one of the most difficult challenges has been to uncover the functional mechanisms of the human brain. Since neuroimaging became the predominant technique in behavioral and cognitive neuroscience, significant advances have been made in the understanding of the human brain. Neuroimaging covers many fields, ranging from the detailed functional architecture of the retinotopically mapped visual cortex to the role of the ventral striatum in emotional learning [1].

Neuroimaging can generally be categorized into two groups: 1) Structural imaging, which addresses the structure of the brain and diagnoses exogenous diseases and injury [2]; and 2) Functional imaging, which diagnoses metabolic diseases and lesions, such as Alzheimer’s disease (AD) [3], [4] and schizophrenia [5], [6]. It first measures some aspect of brain activity, healthy or pathological. Then detect differences when comparing images from healthy control subjects with those of patients. Brain imaging is also used for neurological and cognitive research, such as age-specific changes [7]–[12] and gender classification [13], [14]. Tonks *et al.* [2] found that children with acquired brain injury (ABI) were identified as less resilient and more depressed and anxious than controls. Costafreda *et al.* [15] used an automated analysis to extract 3D hippocampal shape morphology, and then applied machine-learning classifications to predict conversion from mild cognitive impairment (MCI) to AD. Yang *et al.* [5] found reduced gray matter volume in the hippocampus and parahippocampal gyrus in murderers with schizophrenia. Giorgio *et al.* [9] found widespread reductions in the gray matter (GM) volume from middle age onwards and detected earlier reductions in the frontal cortex. Robinson *et al.* [16] proposed a machine-learning-based approach to recognize subjects based on their approximated structural connectivity patterns and to classify subjects of different ages. Tian *et al.* [17] found that compared with females, males have a higher normalized clustering coefficient in the right hemispheric network but a lower clustering coefficient in the left hemispheric network, suggesting a gender-hemisphere interaction.

All the studies described above have been achievements in behavioral and cognitive neuroscience. Such studies almost always involve group analysis (or classification) that identifies population differences by extracting features that represent the key difference between groups [16]. Most of the studies are based on normalization, which has been demonstrated to be useful in many areas, both in research and in clinical settings [18]. For example, in functional imaging studies, normalization of the images is useful to put in line brain architecture from different subjects and determine what happened generally over individuals [19]. The questions in which we are interested are as follows: (1) Is each brain distinct from every other brain? (2) Does normalization reduce the diversity of the brain? The brain coordinates movements, thoughts, and feelings and makes each person distinct. Thus, these issues are important for a deeper understanding of brain research. However, to the best of our knowledge, there are no studies on the diversity (identifying the differences between individuals or identity authentication) of the human brain after normalization. To further our understanding of the mechanisms of brain function, it is desirable to study the diversity of the brain.

The present paper analyzes the diversity of the human brain after normalization for the first time. Using the brain for identity authentication (recognition, similar to face recognition [20], fingerprint recognition [21], or iris recognition [22]), we identified individual differences not only qualitatively but also quantitatively. Our approach first normalized the brain images and produced estimates of the intensity at each voxel. A pixel-to-pixel matching technique was then applied to investigate individual differences in the whole brain. The approach was applied to a database of 150 subjects to evaluate the diversity of the brain based on recognition (identity authentication). The experimental results indicated the individual differences of brain images. We found that the individual differences in using the brain for recognition are as meticulously distinct as those of other biometric features, which confirmed the diversity of brains after normalization.

## Materials and Methods

### Ethics Statement

Approvals for public sharing were obtained from all of the subjects [23], [24].

### Modality Selection

The first step of brain recognition is brain acquisition. There are great developments in various neuroimaging techniques, such as positron emission tomography (PET), computed tomography (CT), electroencephalograph (EEG), magnetoencephalograph (MEG), optical imaging (OI), and magnetic resonance imaging (MRI). These technologies can generally be classified into two categories: invasive techniques and non-invasive techniques. Invasive techniques, such as OI, cannot be applied to healthy humans, even though the technique has a very high spatial resolution. Though MEG technique has a high temporal resolution, it is very expensive. EEG has a high temporal resolution, but it has a significantly lower spatial resolution. Compared with PET, MRI has both high spatial and high temporal resolution, and it does not require radioactive contrast medium, making it non-invasive. Though CT does not rely on radioactive contrast medium, it does use X-ray, which can be harmful.

MRI is a non-invasive brain imaging technique that has been utilized in brain research since the early 1990s [25]. MRI was first introduced in the early 1980s. Since then, it has grown rapidly and become one of the most important brain imaging techniques. The increasing popularity of MRI is due to two characteristics. The first characteristic is that it has no known harmful side effects making it a very patient-friendly and widely-accepted technique. Secondly, it produces images with very high anatomical resolution and specificity especially for soft tissues. Due to its high resolution and non-requirement of radioactive contrast medium, we chose MRI as the modality for this study.

The features that are used for classifications are either structural characteristics or functional properties of the brain [26]. For structural neuroimaging studies, various approaches, including a deformation field for registration [27], a map of cortical thickness [28], and a map of gray matter membership [29], have been employed for classification. For functional neuroimaging studies, a brain activation map [30], a map of regional homogeneity in resting fMRI [31], and brain networks [32] have been used to distinguish patients with mental disorders. Compared with functional brain measures, structural MRI images are more stable and easy to use for individual analysis [29]. Thus, this study adopted structural MRI images for analysis.

### Imaging Protocol

**Figure 1:**
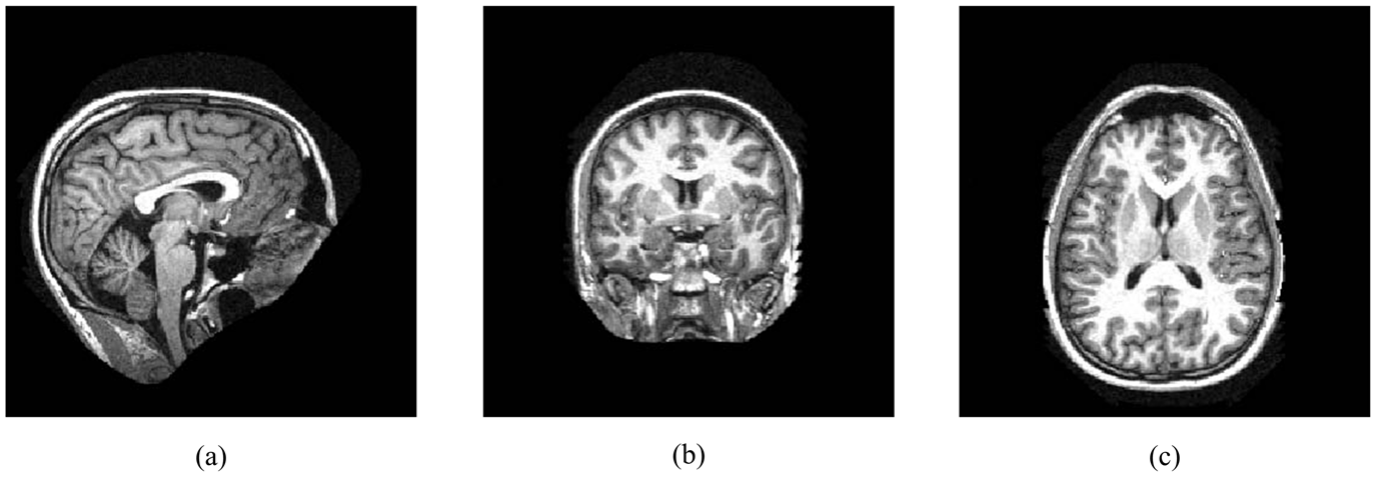
An example of an MR image in the OASIS database. (a) A sagittal section, with the front (anterior) of the head at the right and the top of the head shown at the top. This orientation shows an image as if the subject were being viewed from the right. (b) A coronal section, with the top (superior) of the head displayed at the top and the left shown on the left. This orientation shows an image as if the subject were being viewed from behind. (c) An axial section, with the front (anterior) of the head at the top and the left shown on the left. This orientation appears as if the subject were being viewed from above.

The data set selected for this study was downloaded from the Open Access Series of Imaging Studies (OASIS) website [33]. For each subject, 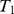-weighted structural magnetization-prepared rapid gradient echo (MP-RAGE) images were obtained with the following parameters: TR = 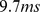, TE = 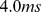, slice thickness = 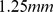, slice number = 128, flip angle = 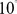, and in-plane resolution = 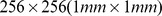. For each subject, 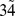 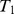-weighted structural images were obtained on a 1.5 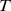 Vision scanner (Siemens, Erlangen, Germany) during a single image session. Figure 1 shows an example of MR images from the OASIS data set. Facial features were removed at the fMRIDC (http://www.fmridc.org) using the Brain Extraction Tool.

### Participants

The subjects were all right-handed and included both men and women. Subsets of the subjects were recruited from the Washington University community and the longitudinal pool at the Washington University Alzheimer Disease Research Center (ADRC). The rest of the subjects were the ADRC’s normal and cognitively impaired subjects, who were recruited primarily through media appeals and word of mouth. Approval for public sharing of the anonymous data was obtained from all of the subjects. For each subject, at least 3 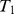-weighted images were acquired per imaging session (visit). Most of the subjects were scanned on several visits at different times.

The receiver operating curve (ROC) plots false acceptance rate (FAR) versus false rejection rate (FRR) [34], and it is often used to evaluate the performance of recognition systems. FRR is defined as the percentage of genuine matching pairs with matching scores below the threshold value, while FAR is defined as the percentage of imposter matching pairs with scores above the threshold. Genuine matching pair indicates that two matching brain images were acquired from the same subject, while imposter matching pair indicates that two matching brain images were scanned from different individuals. In the OASIS data sets, there are many subjects that were scanned on several separate visits (the visits are separated by a time interval: for *OAS1*, this interval is approximately 90 days [23], whereas, for *OAS2*, it is longer than one year [24]). This characteristic is very important for evaluating the performance of identity authentication as the database can generate genuine matching with the characteristic, which is why the OASIS data sets are perfectly suited to this study.

Let 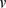 indicates the number of visits for one subject, and 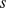 denotes the number of scans generated in each visit. Then the combination number of selecting two visits is 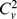. And for each two visits, the number of genuine matching pairs is 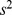. Thus the number of genuine matching pairs for one subject is calculated as
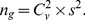
(1)


Let 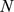 denotes the number of subjects who were scanned at 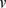 visits, and the total number of genuine matching pairs is
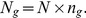
(2)


For *OAS1*, there are only 20 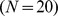 subjects who were scanned for 2 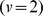 visits, and thus the genuine matching number is very small. In contrast, all 150 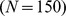 subjects in *OAS2* were scanned for 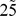 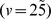 visits; therefore, there are more genuine matching pairs in *OAS2* than in *OAS1*. Thus, we chose *OAS2* as the data set for this study. Another reason for choosing *OAS2* is that the interval between visits is longer than one year for *OAS2*, whereas the interval for *OAS1* is shorter than 90 days. A longer interval yields a stronger ability of the data set to evaluate the robustness and steadiness of the authentication system.

### Normalization

Normalization is ubiquitous and important in many aspects of image analysis [19]. For example, in neuroimaging, the realignment of a time-series of scans from the same subject (correcting for movement) is necessary for voxel-based analysis of time-dependent changes [35]. To average signals from functional brain images of different subjects, it is necessary to register the images together [36]. This issue is the current interest in the analysis of functional magnetic resonance imaging (fMRI) time-series. Inter-subject averaging with change distribution analysis or statistical parametric mapping also requires the images to be transformed into a standard stereotactic space [35]. This procedure is known as normalization. A fundamental advantage of normalization is that activations can be reported according to a set of meaningful Euclidian coordinates within a standard space [37].

Normalization is often performed by determining the correspondence between images. At its simplest, image normalization involves estimating a smooth, continuous mapping between the points in one image and those in another. The relative shapes of the images can then be determined from the parameters that encode the mapping (spatial transformation) [38]. There are many ways of modeling such mappings, and they can generally be classified into two broad categories [39]:

- *rigid-body* Transformations that preserve the distance between all points in the image are called rigid-body transformations. These transformations consist of shift and rotation, which are equivalent to a change from one Cartesian system of coordinates to another one. Rigid-body transformation is a specific case of affine transformation, which allows for a global change of scale.
- *nonrigid* Transformations that map straight lines to curves are referred to as nonrigid transformations. In contrast to rigid-body transformations (in which the constraints are explicit), the constraints for nonrigid warping are more arbitrary.

A rigid-body transformation may be perfectly valid for realignment but not for spatial normalization of an arbitrary brain into a standard stereotaxic space [37]. The objective of this study is not to achieve a high accuracy of realignment, although a more accurate realignment leads to better performance of the brain matching and identity authentication. In contrast, in this study, we mainly analyzed the diversity of brains after normalization. Thus, we used nonrigid transformation, which can spatially normalize an arbitrary brain into a standard stereotaxic space.

There are many different nonrigid transformation models. In general, these models can be divided into two categories [38], [40]:

- A small-deformation framework does not necessarily preserve topology, although if the deformations are relatively small, then the topology may still be preserved.
- A large-deformation framework generates deformations (diffeomorphisms) that have a number of elegant mathematical properties, such as enforcing the preservation of topology.

DARTEL (diffeomorphic anatomical registration by exponentiated Lie algebra), which is a large-deformation technique, has been widely used for normalization since its first establishment. DARTEL has the advantage, relative to the small-deformation approach, that the resulting deformations are diffeomorphic and easily invertible and can be rapidly computed [38]. DARTEL is also more accurate than conventional SPM normalization [41]. When good-quality anatomical MRI scans are available, the DARTEL approach is now generally recommended [42]. For the above reasons, we chose DARTEL as the method of normalization for this study.

The normalization was performed using SPM8 [43], and it consisted of three steps. First, the new segment procedure was used to segment the MRI images into six partitions, including gray matter (GM), white matter (WM), cerebrospinal fluid (CSF), and three other background partitions based on a modified mixed-model cluster analysis technique. The new segment procedure is generally more robust than using the “segment” button. Next, a template was generated from the entire image data set using the diffeomorphic anatomical registration by exponentiated Lie algebra (DARTEL) technique [38]. Finally, the GM images were spatially normalized to the template that was created in the second step and then smoothed by an isotropic Gaussian filter with an 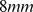 full-width half-maximum (FWHM) kernel.

### Matching

Matching methods can be mainly divided into three classes [34]: (1) algorithms that use the image pixel values directly; (2) algorithms that use low-level features, such as edges and corners; and (3) algorithms that use high-level features. The drawback of high-level matching methods is that high-level features first need to be extracted and identified, which is a rather difficult task. Low-level matching is often used in circumstances in which the object to be recognized is well structured, such as fingerprints that have *minutiae* [21] as their stable feature. However, normalized brain images do not have such steady point features.

The human brain consists of white matter, gray matter and cerebrospinal fluid. The gyrus is distributed among white matter and gray matter. The gray intensity of each brain is different [44]. To recognize different persons, we can compare differences in brain gray intensity. Thus, we treat brain matching as a problem of pixel-level matching. Compared with the other two methods, pixel-level matching methods have more potential information from the images than methods that extract features for matching.

To compare two images, the first step is to align the images. In this study, we have finished the alignment step in the normalization stage (see section “**Normalization**”). Let 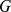 denote the normalized gray matter image of the input brain image 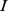. In the matching step, the difference between two aligned gray matter images, 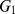 and 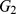 (normalized from the two MR images, denoted as 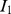 and 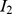, respectively), is computed as below:
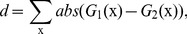
(3)where x is all of the possible three-dimensional coordinates corresponding to the gray matter images. We used the segmented image; the each voxel shows the proportion/probability of it being GM. This procedure makes the matching robust to noise, smear, lesion, and other effects. A smaller *d* indicates a higher probability that the two MR images come from the same subject. The final score *s* is normalized from *d* by
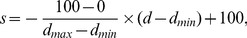
(4)in which 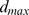 and 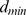 represent the maximum and minimum value of all of the differences 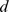, respectively. Equation (4) normalized the scores to the interval [0, 100].

### Experimental Protocol

The experiments were conducted on *OAS2*. We can generate genuine matching using images obtained in the same session from the same subject (denoted as ‘Same-Visit’); or using images from the same subject but from different visits (denoted as ‘Different-Visit’).

## Results

### Experimental Results

**Figure 2:**
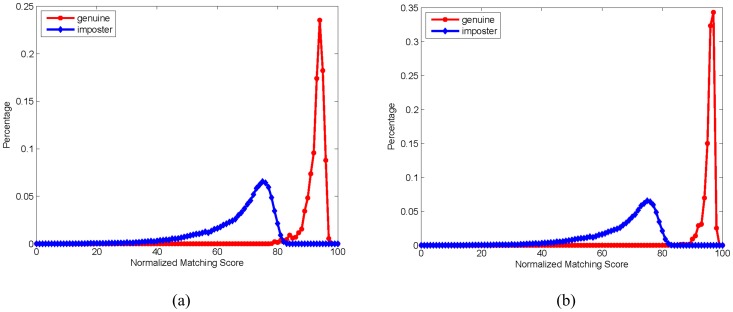
The distributions of correct and incorrect matching scores. The vertical axis represents the distribution of matching scores as a percentage. (a) The distribution of matching scores in the ‘Same-Visit’ experiment. (b) The distribution of matching scores in the ‘Different-Visit’ experiment.

The distributions of normalized genuine and imposter matching scores are shown in Figure 2. It can be observed from this figure that two peaks exist in the distribution of matching scores. One peak is located at a value near 75, corresponding to the imposter matching scores. The other pronounced peak resides at a value of 95 and is associated with the genuine matching scores. This result indicates that the proposed scheme is capable of differentiating brains at a high rate of accuracy by selecting an appropriate value of the threshold. Figure 2 shows that the performance of the ‘Same-Visit’ experiment is better than that of the ‘Different-Visit’ experiment, as the genuine scores of the ‘Same-Visit’ experiment are higher and more concentrated than those of the ‘Different-Visit’ experiment.

**Figure 3:**
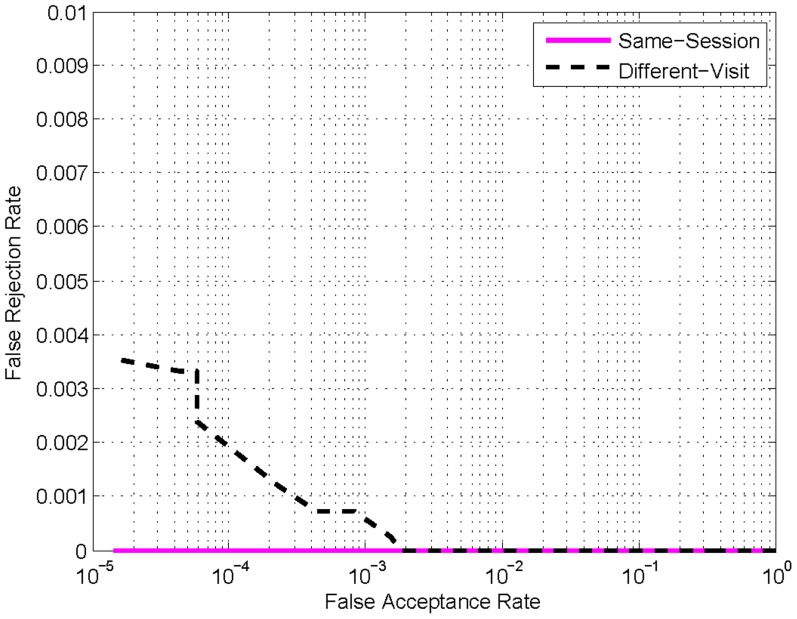
The ROCs of the matching experiments. The solid line (pink line) represents the ‘Same-Visit’ experiment, while the dashed line (black line) represents the ‘Different-Visit’ experiment.

Figure 3 shows the receiver operating curves (ROC) plotting FAR versus FRR of the ‘Same-Visit’ experiment and the ‘Different-Visit’ experiment. The results show that the performance of the ‘Same-Visit’ experiment is much better than that of the ‘Different-Visit’ experiment. The equal error rates (EER, the point when FAR is equal to FRR) are 0.0% and 0.072% for the ‘Same-Visit’ and the ‘Different-Visit’ experiments, respectively. This discrepancy illustrates that the matching pairs (genuine and imposter) in the ‘Same-Visit’ experiment are more distinct. Furthermore, it can be judged from Figure 2 that the matching pairs (genuine and imposter) in the ‘Same-Visit’ experiment can be absolutely separated by a well-defined threshold, such as 85. Specifically, the maximum imposter matching score in the ‘Same-Visit’ experiment is 83.46, while the minimum genuine matching score is 86.03. Thus, the matching pairs (genuine and imposter) in the ‘Same-Visit’ experiment can be absolutely separated by any threshold value between 83.46 and 86.03, which is why we can obtain an EER of 0.0% for the ‘Same-Visit’ experiment.

To further evaluate the diversity of brains after normalization, we also conducted an identification experiment. An identification system is a 1-to-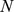 (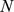 is the number of brains or subjects in the database) matching system. When an input brain image is given, the identification system compares the input image with all of the brain images in the database and returns the image (or the ID of the subject) with the highest matching score. If the returned brain image and the input image are from the same brain (subject), it is called *right-identification*. The *identification ratio* is defined as the ratio of *right-identification* inputs to all input brain images. In this study, we used the second scan in the first session (visit) of every subject to establish the database and treated the second scan in the second session (visit) of every subject as the inputs. We obtained an exciting result: a 100% *identification ratio* was achieved in the testing database.

## Discussion

It is a consensus that human brains are different from each other. In this study, we proposed to measure the individual differences in the human brain for the first time. Using brain images for identity authentication, we found that the individual differences are extremely discriminative. The EER of the ‘Same-Visit’ experiment is incredibly small, actually being equal to 0.0%. This result indicated that each subject’s brain is clearly different from all others. The proposed matching strategy is based on normalization, which is a pre-processing stage that is widely adopted in neuroimaging analysis. Thus, the experimental results confirmed that the normalization stage retains almost all of the information and features of brain images.

Normalization is the process of mapping a single subject’s brain image into a standard space [37]. It is a widely used pre-processing stage in neuroimaging. In functional imaging studies, normalization of the images is useful for determining what happens generically across individuals. One advantage of using normalized images is that inter-subject analysis can be conducted according to their Euclidean co-ordinates within a standard space. The other advantage of normalization is that it increases the sensitivity in detecting activations in brain imaging [19]. Normalization is extremely widely used, and the studies of the performance of normalization can be categorized into three kinds: permutation tests, one-way ANOVA tests, and indifference-zone ranking [41]. However there is no study or report on the individuality of the brain after normalization by identity authentication, to the best of our knowledge. This study confirmed the diversity of the brain by employing brains for identity authentication. The result that the *identification ratio* can reach 100% provides evidence that normalization can preserve the diversity of the brain. Thus, normalization is an appropriate pre-processing stage due to the advantages mentioned above.

The reduction in performance of the ‘Different-Visit’ experiment compared with the ‘Same-Visit’ experiment is primarily caused by two reasons. First, noise stemming from the electronics of the MR system makes the scanning different for each visit. Second, the external environment is not exactly the same for each session. These two variables result in several variations in the biometric patterns of the brain images from the same subject (“intra-class variability”). Thus, the genuine matching scores in the ‘Different-Visit’ experiment are lower than those in the ‘Same-Visit’ experiment, which reduces the discriminative ability of genuine matching from imposter matching in the ‘Different-Visit’ experiment. However, the performance of the ‘Different-Visit’ experiment is still very high compared with other biometric technologies.

Table 1 shows the performance of common biometric technologies. We selected state-of-the-art technologies, and the scales of the corresponding databases are almost equivalent. Table 1 shows that the EER of the ‘Different-Visit’ experiment is as low as those of other biometric techniques, which illustrates that brain recognition is robust as well as steady. One significant advantage of the proposed brain recognition system is its ability to achieve a 0.0% or near 0.0% EER, i.e., a clear separation of genuine and imposter distributions. This study presents the first exploration of utilizing the brain for authentication, and we believe that the brain will be one of the members of biometric technologies in the future.

**Table 1:** Statistics of EER for different biometric technologies.

Technology	*N_s_*	*N_g_*	*N_i_*	EER(%)
Face [46]	100	1600	158400	2.22
Fingerprint [47]		27720	87990	0.118
Iris [48]	460			0.086
Palmprint [49]	250	7500	4491000	0.021
Brain (proposed)	150	4212	11175	0.072

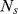
 – the number of subjects (individuals) in the corresponding database.

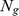
 – the number of genuine matching pairs.

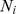
 – the number of imposter matching pairs. A blank in the table indicates that the corresponding item was not reported.

To compare with DARTEL, we have conducted an experiment using another well-known registration method. This segmentation method was done using the SPM8 MATLAB software [42] and the VBM8 toolbox (http://dbm.neuro.uni-jena.de). The EER are 0.3% and 2.1% for the ‘Same-Visit’ and the ‘Different-Visit’ experiments, respectively. Though this is roughly acceptable in a preliminary biometric system, its performance is not as good as DARTEL. It illustrates that different registration methods preserve the diversity of brain to different level, and the matching performance varies by different registration methods. A better registration method results in a better matching performance.

Although our method performs well, several issues remain. First, although the EER of ‘Same-Visit’ experiment equals to 0.0%, the gap between maximum imposter matching score (83.46) and the minimum genuine matching score (86.03) is very small. Second, the identification ratio reaches 100%, but the database contains only 150 subjects (1366 images), and a larger database should be established to evaluate the performance of brain recognition in the future. Third, though the recognition ratio is very high, the acquirement of brain is still not very convenient for users and it is also not as cheap as other biometric equipment.

To conclude, the approach used in this study analyzed the individuality of brain after normalization. This paper proposed to use the brain for identity authentication. Biometric recognition technologies are generally based on the diversity of biological (e.g., face, fingerprint, and iris) traits [45]. Compared with these biometric technologies, the proposed brain recognition technique achieved similar recognition ability. Thus, the result confirmed the diversity of the brain after normalization. The proposed approach used the whole brain for matching, and a more detailed study analyzing the diversity of different regions within the brain will be left for future work.
